# A knowledge-enhanced transform-based multimodal classifier for microbial keratitis identification

**DOI:** 10.1038/s41598-023-36024-4

**Published:** 2023-06-02

**Authors:** Jianfeng Wu, Zhouhang Yuan, Zhengqing Fang, Zhengxing Huang, Yesheng Xu, Wenjia Xie, Fei Wu, Yu-Feng Yao

**Affiliations:** 1 School of Medicine, Zhejiang University, Hangzhou, Zhejiang Province, 31002 China; 2 College of Computer Science and Technology, Zhejiang University, Hangzhou, Zhejiang Province, 31002 China; 3 Department of Ophthalmology, Sir Run Run Shaw Hospital, Zhejiang University School of Medicine, Hangzhou, Zhejiang Province, 310016 China; 4 Key Laboratory for Corneal Diseases Research of Zhejiang Province, Hangzhou, Zhejiang Province, China

**Keywords:** Corneal diseases, Machine learning

## Abstract

Microbial keratitis, a nonviral corneal infection caused by bacteria, fungi, and protozoa, is an urgent condition in ophthalmology requiring prompt treatment in order to prevent severe complications of corneal perforation and vision loss. It is difficult to distinguish between bacterial and fungal keratitis from image unimodal alone, as the characteristics of the sample images themselves are very close. Therefore, this study aims to develop a new deep learning model called knowledge-enhanced transform-based multimodal classifier that exploited the potential of slit-lamp images along with treatment texts to identify bacterial keratitis (BK) and fungal keratitis (FK). The model performance was evaluated in terms of the accuracy, specificity, sensitivity and the area under the curve (AUC). 704 images from 352 patients were divided into training, validation and testing set. In the testing set, our model reached the best accuracy was 93%, sensitivity was 0.97(95% CI [0.84,1]), specificity was 0.92(95% CI [0.76,0.98]) and AUC was 0.94(95% CI [0.92,0.96]), exceeding the benchmark accuracy of 0.86. The diagnostic average accuracies of BK ranged from 81 to 92%, respectively and those for FK were 89–97%. It is the first study to focus on the influence of disease changes and medication interventions on infectious keratitis and our model outperformed the benchmark models and reaching the state-of-the-art performance.

## Introduction

Microbial keratitis (MK) is one of the most common corneal diseases and a major cause of visual impairment^1–3^. The distribution of MK varies from country to country due to climate, contact lens use, socioeconomic status and accessibility of health services^2,4,5^. With the prevalence of corneal contact lenses, the incidence of bacterial keratitis (BK) and fungal keratitis (FK) is increasing^6^.The management of FK and BK is challenging, surgical intervention is usually required at late stage, and poor visual outcomes are usually encountered^2,7,8^. Hence, early diagnosis is essential to avoid devastating consequences that threaten vision.

However, it is not easy to diagnose FK and BK at an early stage. It has been reported that correctly differentiating between BK and FK is a challenging process even for trained corneal experts and is often misdiagnosed in more than 30% of the cases^9^. When ophthalmologists are unable to ensure the pathogens of keratitis, they usually use empirical therapy without microbiological results until culture results are available^10^. The rationale for empirical treatment is based on the assumption that most cases of bacterial keratitis will respond to modern broad-spectrum antibiotics^11,12^. And some ophthalmologists treat corneal infections empirically with the newer fluoroquinolone antibiotics, even without the procedures of Gram staining and culture^13^. Yet the failure of treatment may increase the likelihood of advancing corneal infiltration and a poor therapeutic outcome^14^ and the time lag between empirical treatment and the appearance of results may let the patients to miss the optimal time to initiate appropriate treatment.

In computer-aided diagnosis, deep learning algorithms with artificial intelligence (AI) are now widely used for medical image recognition and making great progress in the field of ophthalmology, such as diabetic retinopathy^15^, age-related macular degeneration^16^, glaucoma^17^, and topography for keratoconus^18^. Until now, few studies have applied deep learning on infectious keratitis (IK) using slit-lamp microscopic images and there is still a major improvement in terms of classifying BK and FK^19–22^. And no reported models have applied multimodal information to improve diagnostic accuracy for keratitis. However, in the real world, there is a lot of disturbance in images and doctors make a judgement based on multidimensional information such as pathological images, medical history and laboratory results. Due to the rapid disease progression of BK and FK, the few visits at beginning basically determined the treatment plan and the patient's prognosis.

Based upon that, we aimed to develop a knowledge-enhanced transform-based multimodal classifier (KTBMC) that employs images in addition to text to improve the prediction and to aid ophthalmologists in diagnosing BK and FK.

## Materials and methods

### Image datasets

The image dataset for this study included 158,931 clinical digital images taken from 15,687 patients with 89 categories of corneal diseases by slit lamp microscopy during the period of October 2004 to 2020 in the Department of Ophthalmology, Sir Run Run Shaw Hospital, School of Medicine, Zhejiang University. The study was approved by the Ethics Committee of Sir Run Run Shaw Hospital, Zhejiang University School of Medicine (Ethical approval code: 20210318-32) and adhered to the ARVO statement on human subjects and the Declaration of Helsinki. The Ethics Committee of Sir Run Run Shaw Hospital, Zhejiang University School of Medicine waived the need for informed consent for patients in this study based on a retrospective design and the privacy protection via delinking personal identification at image and data analysis.

In the dataset, images taken from patients whose initial treatment was anti-microbial therapy, including BK and FK, were selected for the training or testing set for algorithmic classification into each infectious category. For each patient, only two images, the initial presentation and the first follow-up, were selected for the dataset. All the images from the patients with corneal infections were annotated with a definite clinical diagnosis that was corroborated by at least one piece of the following evidence: ① the progression of the corneal infection was influenced and terminated by diagnostic pertinent single-drug or combined-drug therapy leading to its ultimate curing; ② pathogen identification of the sample from the infection site: either confirmed by sample smear under microscopic examination or organism culture.

Patients were excluded if they had mixed bacterial and fungal infections; corneal perforation; no documented slit-lamp images; poor-quality or fluorescein-staining images; or the presence of other corneal diseases, such as viral keratitis, Acanthamoeba keratitis, marginal keratitis, corneal dystrophy or degeneration, chemical burn, mucous membrane cicatricial pemphigoid, or bullous keratopathy.

The final dataset contained 704 images from 352 patients for this study. The training set consisted of 262 randomly selected images of BK and 296 images of FK from 279 patients. And the training set was randomly divided into a training set and a validation set in the ratio of 4:1. The testing set consisted of 72 randomly selected images of BK and 74 images of FK from 73 patients.

### Treatment text datasets

Information on the course of all patients' illnesses and their medication history from their initial visit was collected in paper and electronic medical records. First we stored all patients' medication records for the initial diagnosis as electronic data by hand, then under the clinician's guidance, we excluded medication unrelated to the treatment of infectious keratitis, such as medication for dry eyes or glaucoma. As treatment text is relatively simple and short, it didn't require much preprocessing. We converted all medication names into lowercase proper names and doses were processed according to a uniform prescription format. Then the words segmented by space were directly fed into the pre-trained Bert to extract embeddings. The final top ten word frequency statistics by space division are shown in the Fig. 2.

Common anti-bacterial drugs include Levofloxacin, Ofloxacin, Cefuroxime Sodium and Amikacin. Depending on the dose, it can be used to prevent and control bacterial infection. Common anti-fungal drugs include Itraconazole, Natamycin, Voriconazole and Amphotericin B. No private information was collected or compromised.

### Knowledge-enhanced transform-based multimodal classifier

**Figure 1:**
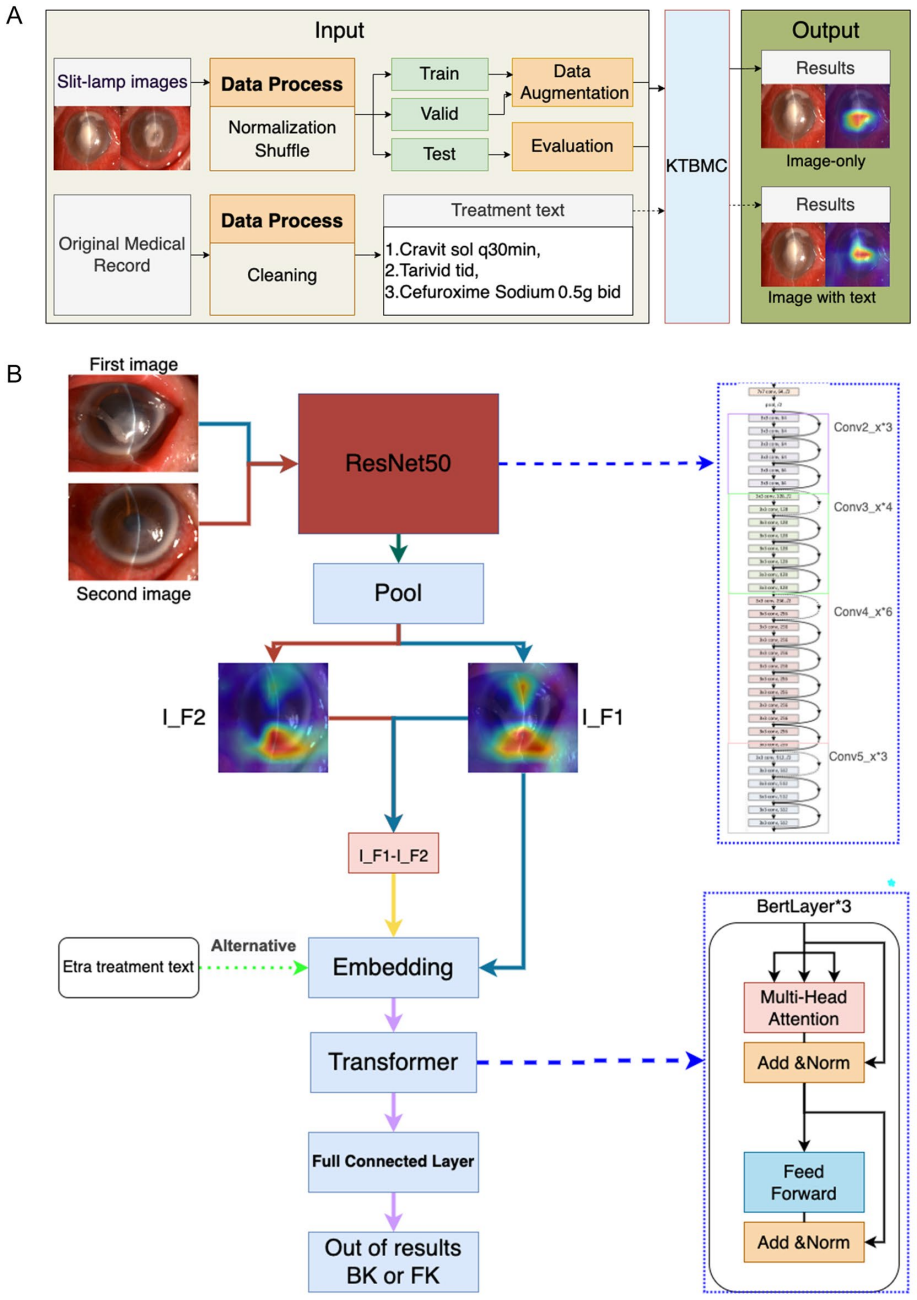
The whole deep learning framework. (**A**) The abstract flow chart for developing the deep learning model. (**B**) The architecture of KTBMC applied ResNet50 to extra image features and used BERT embeddings to contact all features to classify. I_F1—First image feature, I_F2—Second image feature.

The knowledge-enhanced transform-based multimodal classifier was based on Convolutional Neural Network (CNN) and BERT^23,24^. The algorithm architecture is illustrated in Fig. 1 (take ResNet50 for example).

It is usually to transfer the final fully connected layer of a pre-trained convolutional neural network, where the output is often the result of pooling over feature maps. Since the transformer can handle an arbitrary number of dense inputs, we try to produce not a single output vector, but N separate image embeddings, unlike in a regular convolutional neural network^23^. In this case, we used a pre-trained ResNet with average pooling (DenseNet with norm5) over the K × M grids in the image, yielding N = KM output vectors for every image. As we input two images at one time, the features of the two images were extracted separately and the first was input into the embedding layer along with the difference between the first and the second. Before being input into the image encoder, all of the images were resized to a resolution of 256 × 256 × 3. Then they are also randomly cropped to a resolution of 224 × 224 × 3 and each of them was normalized into (0,1), which enabled the model to converge more quickly and steadily.

We used four CNNs (i.e., ResNet50, ResNet152^25^, DenseNet121 and DenseNet169^26^) as our model image encoder. We pre-trained these models on a four-categorical classification dataset containing 24,818 images of amoeba keratitis, BK, FK, and herpes simplex keratitis. And we used the pre-trained 3-layer 768 dimensional base-uncased model for BERT, trained on English Wikipedia^23^.

The architecture takes embeddings as input, where we can put image embeddings as well as text embeddings. Since BERT is an extremely large-scale model and our dataset is too tiny to train it, we just trained the final classification layer and froze the embedding parameter settings. The experiment hyperparameter configuration was showed in the supplementary file (Table S1).

To compare the performance of our models, we applied four CNNs on the same data set with a single image as input.

### Performance interpretation and statistics

For visualizing heat maps, the gradient-weighted class activation mapping (GradCAM) technique^27^, in which the model’s attention scores are computed according to the calculation of the gradients before the embedding layer, was used to plot the heat map of the model. Receiver operating characteristic (ROC) curves were illustrated to discriminate between BK and FK, and AUC was measured. Youden’s index was used by the ROC curve to obtain the sensitivity and specificity. The accuracy of the model was further calculated. Statistical analysis was performed with R (R Core Team, 2022) and figures were produced using the package ggplot2.

## Results

### Patient distribution and characteristics

**Figure 2:**
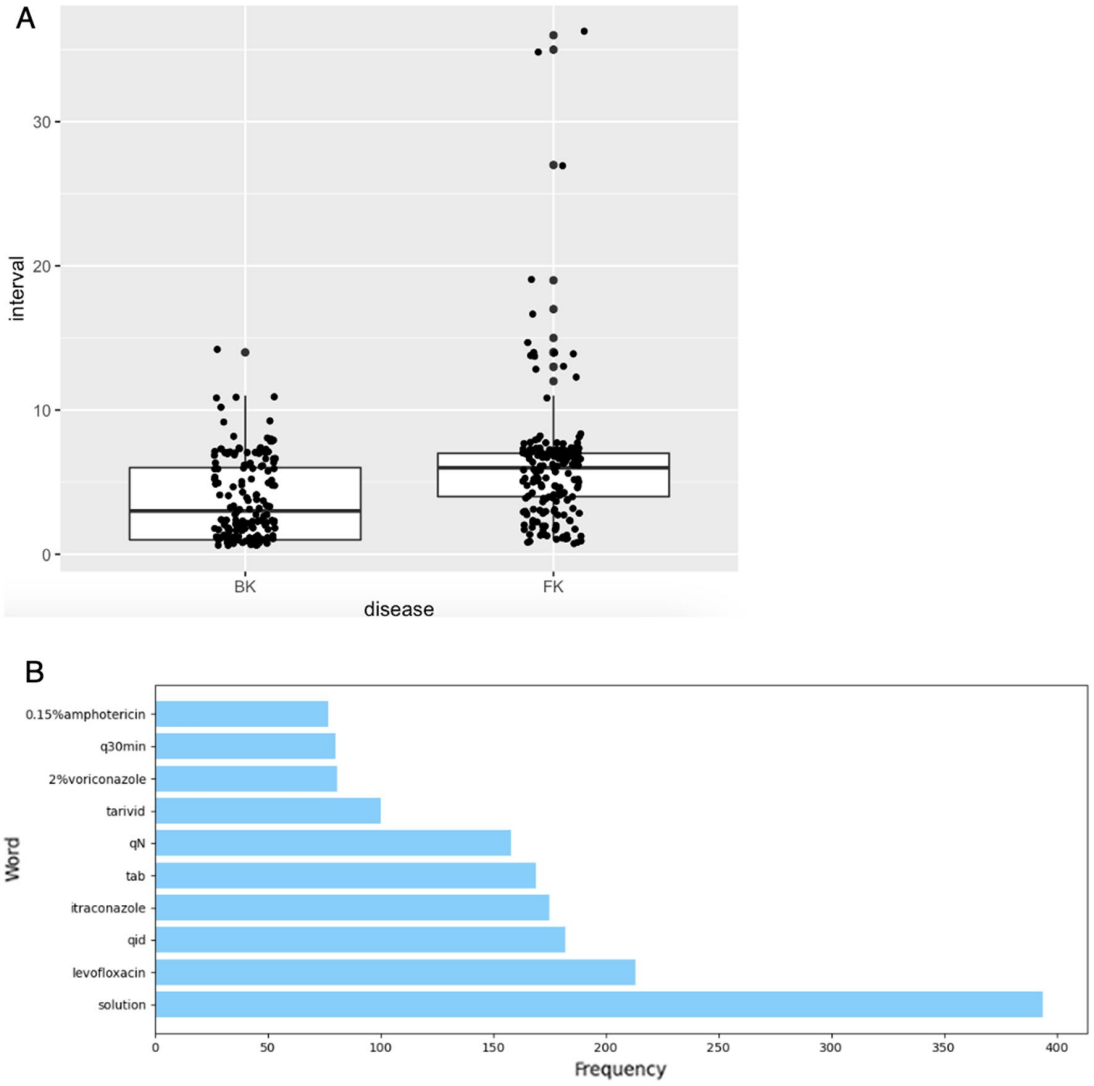
(**A**) Boxplot of interval of days distribution, (**B**) the final top ten results of the text word frequency statistics.

A total of 352 patients (216 males and 136 females) with 704 images were included. The average patient age was 53.6 ± 11.5 years. The distribution and characteristics of the patients are shown in Table 1. Because we choose only two images for one patient and input all into the model at the same time, the days between the initial presentation and first follow-up were also a very important variable. And we also plotted a boxplot with jittered points of the variable to visually compare the distribution of the variable before the two disease types and results of the text word frequency statistics (Fig. 2).

**Table 1:** Distribution of patients in the train and test datasets.

Characteristics	Total (n = 352)	Train (n = 223)	Validation (n = 56)	Test (n = 73)
Male, n (%)	216(61.4)	140(62.8)	37(66.1)	39(53.4)
Age, years*	53.6 ± 11.5	52.5 ± 12.2	55.4 ± 9.8	55.7 ± 10.6
	*Patients, n (%)/photos, n*			
Bacteria	167(47.4)/334	105(29.8)/210	26(7.4)/52	36(10.2)/72
Fungal	185(52.6)/370	118(33.5)/236	30(8.5)/60	37(10.5)/74
	Days of interval (days)*			
Bacterial	3.7 ± 2.4	3.3 ± 2.1	4.7 ± 2.4	4.2 ± 2.7
Fungal	6.2 ± 2.7	6.1 ± 2.7	6.0 ± 2.0	6.6 ± 3.1

*Continuous variables presented as the mean ± standard deviation, n: number, days of interval: time between initial presentation and first follow-up.

### Performance of backbone

We chose four CNNs with a single image as the input to serve as the benchmark for our experiments, and the results are presented in Table 2. And ResNet50 performed best with an average accuracy of 0.86.

**Table 2:** Performance of benchmark.

CNN	Validation	Testing	Average accuracy
ResNet152	0.82	0.66	0.74
ResNet50	0.88	0.83	0.86
DenseNet121	0.68	0.58	0.63
DenseNet169	0.75	0.59	0.67

### Performance of KTBMC

Owing to the flexibility of transformers, we can change the different inputs to test the performance of our model. Details regarding the accuracy, sensitivity and specificity of all of the models are presented in Table 3. The average accuracy of two images as input ranged from 88 to 91%. When adding extra treatment text, the best average accuracy, and accuracy of BK and FK increased to 97%, 92%, and 95% severally with DenseNet121 and the remaining models all had improved in accuracy. It indicated extra treatment did help with model classification. And our dataset is so small, it is indeed easy to overfit. The loss results for KTBMC with different input and CNN in Supplementary Fig. S1.

**Table 3:** Performance of different input and CNN.

Input/CNN	Validation	Testing	Average accuracy	BK	FK	Sensitivity	Specificity	PPV	NPV
						[95% confidence interval]			
*Image*									
ResNet152	0.91	0.88	0.90	0.83	0.92	0.92 [0.77,0.98]	0.83 [0.67,0.93]	0.85 [0.69,0.94]	0.91 [0.75,0.98]
ResNet50	0.89	0.86	0.88	0.83	0.89	0.89 [0.74,0.96]	0.83 [0.66,0.93]	0.85 [0.69,0.94]	0.88 [0.72,0.96]
DenseNet121	0.93	0.88	0.91	0.83	0.92	0.92 [0.77,0.98]	0.83 [0.67,0.93]	0.85 [0.69,0.94]	0.91 [0.75,0.98]
DenseNet169	0.93	0.86	0.90	0.81	0.92	0.92 [0.77,0.98]	0.81 [0.63,0.91]	0.83 [0.67,0.92]	0.91 [0.74,0.98]
*Image* + *text*									
ResNet152	0.93	0.92	0.93	0.86	0.97	0.97 [0.84,1]	0.86 [0.7,0.95]	0.88 [0.73,0.95]	0.97 [0.82,1]
ResNet50	0.91	0.92	0.92	0.86	0.97	0.97 [0.84,1]	0.86 [0.7,0.95]	0.88 [0.73,0.95]	0.97 [0.82,1]
DenseNet121	1	0.93	0.97	0.92	0.95	0.95 [0.8,0.99]	0.92 [0.76,0.98]	0.92 [0.78,0.98]	0.94 [0.79,0.99]
DenseNet169	0.98	0.89	0.94	0.89	0.89	0.89 [0.74,0.96]	0.89 [0.73,0.96]	0.89 [0.74,0.96]	0.89 [0.73,0.96]

**Figure 3:**
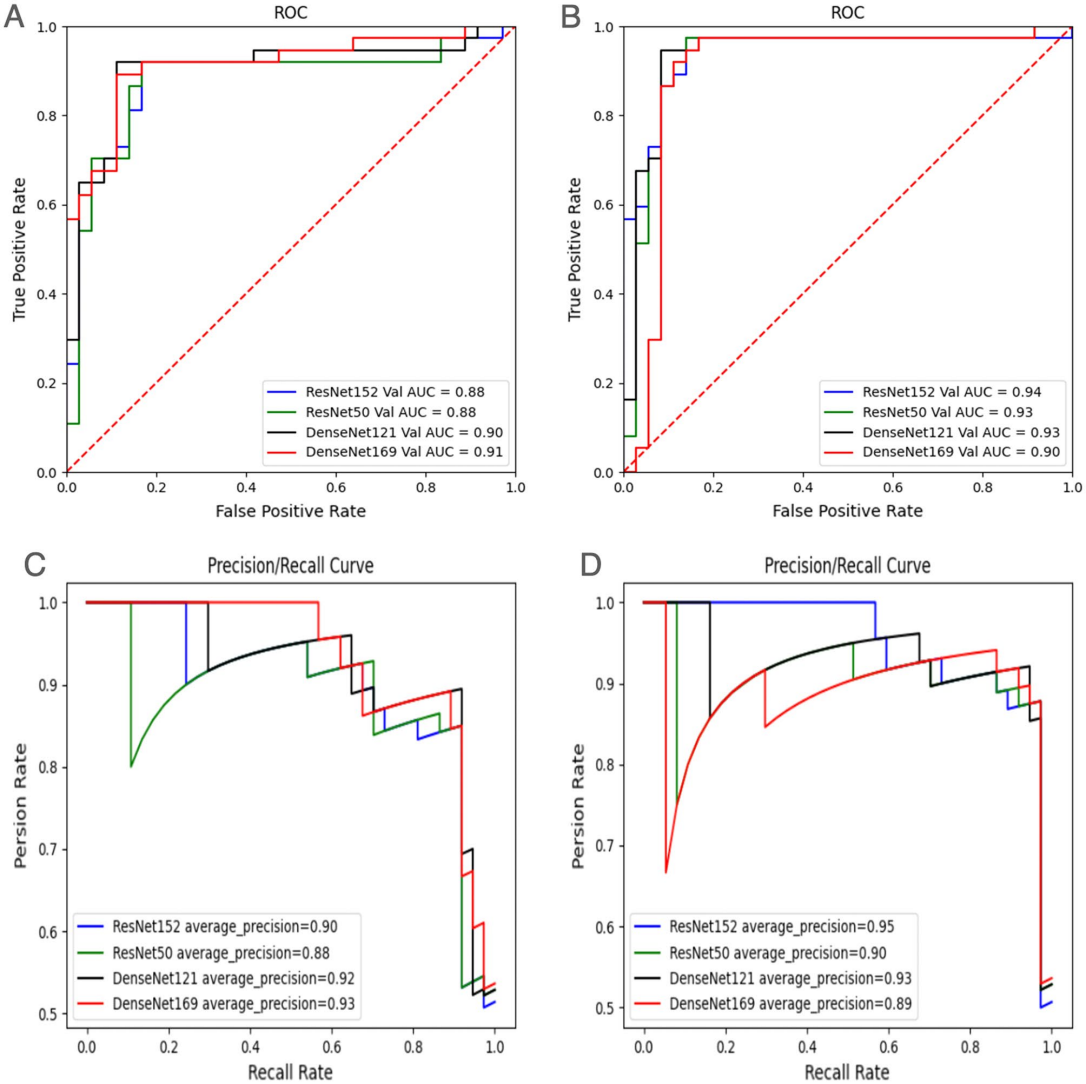
Receiver operating characteristic curves and Precision/Recall curves of KTBMC for four image encoders (**A**) ROC without treatment text. (**B**) ROC with treatment text. (**C**) PR without treatment text. (**D**) PR with treatment text.

ResNet152 was the best model that achieved an AUC of the ROC curve of 0.94(95% CI [0.92,0.96]) for both BK and FK. And ResNet152 was also best in a precision-recall curve with an average precision of o.95 (Fig. 3).

### Instance analysis

**Figure 4:**
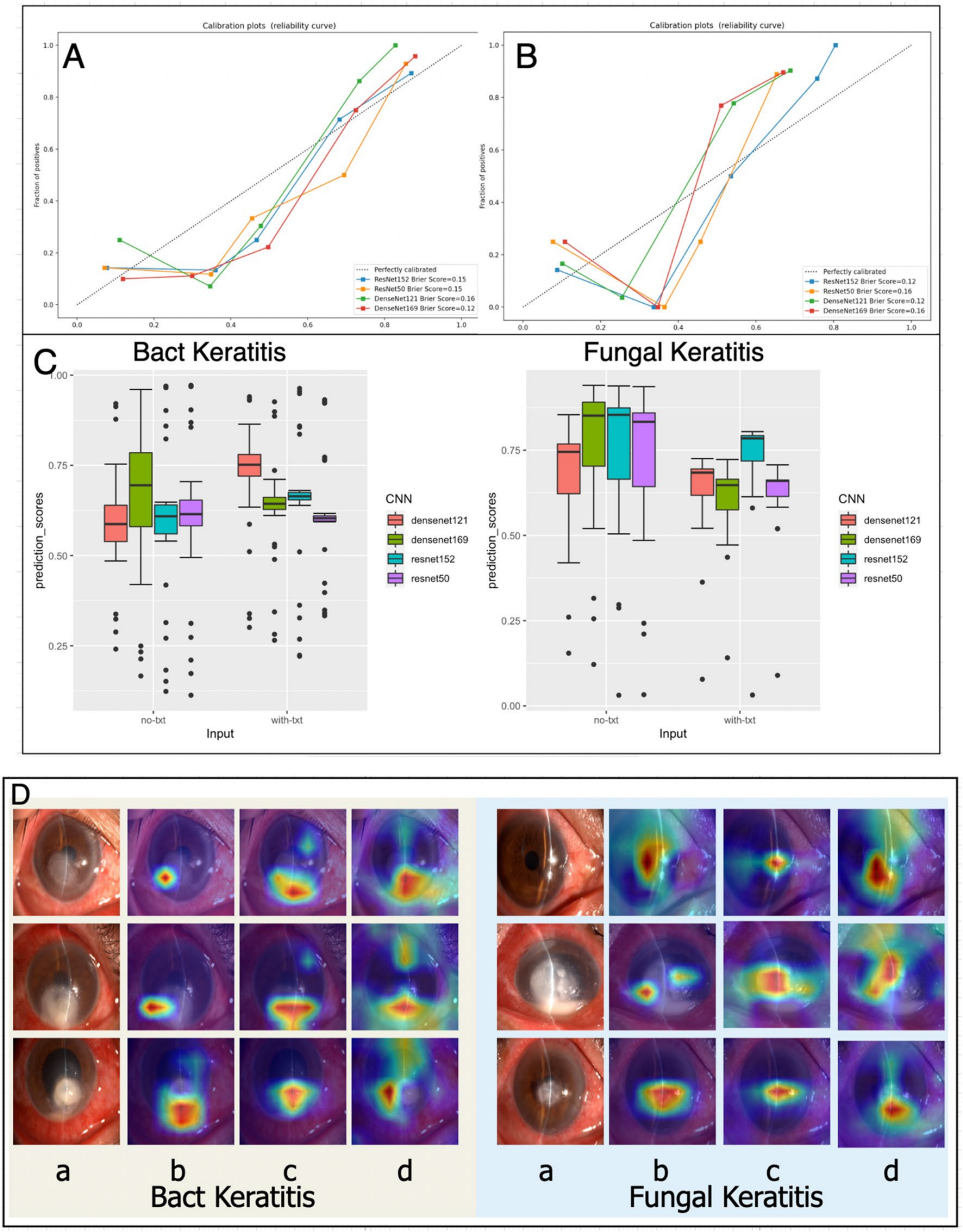
Model calibration and Brier Score of KTBMC (**A**) without treatment texts and (**B**) with treatment texts. (**C**) Boxplot of prediction scores of KTBMC output. (**D**) Heat maps generated by models that were hard to correctly classify. Column (**a**): original images. Column (**b**): heat maps generated by KTBMC without treatment texts. Column (**c**): heat maps generated by KTBMC with treatment texts. (**d**): heat maps generated by ResNet50. * No-txt: input without treatment texts. With-txt: input with treatment texts.

We printed all prediction scores after SoftMax and had some discoveries. As we can see from Fig. 4 that BK was harder to classify than FK on all CNNs with a *P* value (*P* < 0.05) when only images were used as input. And after adding treatment texts, the prediction scores of BK markedly improved on DensNet121 with a *P* value (*P* < 0.001). Correspondingly, the other prediction scores had no difference.

Model calibration was used to assess whether the model output was representative of the true probabilities. And ResNet152 performed best with the minimum Brier Score of 0.12 (Fig. 4).

We selected some samples that were at the classification boundary, and the heat map generated with Grad-CAM for model visualization is presented in Fig. 4.

## Discussion

In this study, we mainly developed a brand-new deep learning model which combined CNN with BERT to improve the accuracy of diagnosis of BK and FK. The model applying slit-lamp images and treatment texts achieved an average accuracy of 97%, and diagnostic accuracies of about 92% and 95% for BK and FK respectively (Table 3), far exceeded the performance of corneal specialty ophthalmologists whose accuracy was up to 76% on FK^28^ and compared to senior attend ophthalmologists with a maximum accuracy rate of 88%^19^. And the sensitivity for detecting keratitis was 95% (95% CI [80%,99%] and the specificity was 92% (95% CI [78%,98%]), which demonstrated the broad generalizability of our model.

Additionally, we selected four CNNs as benchmarks to compare KTBMC’s ability and our model far exceeded them (Table 2). And our models were also performed using different CNNs as image encoders. All CNNs had similar performance (Table 3). It was probably due to the powerful model performance of the BERT, so KTBMC did not over rely on CNNs.

To make the output of our model interpretable, heat maps were generated to visualize where the system attended for the final decisions (Fig. 4D). And we also chose ResNet50 which performed best to produce the heat maps for comparison. It was pointed out that CNN with a single image as input would focus on regions outside the cornea, such as the eyelid or conjunctiva if there is no image crop^20^. From Fig. 4D, ResNet50 did focus on areas outside the lesion and we concluded that our model was able to distinctly focus and learn the features from dominant lesions like the epithelial defect, oedema and deep stromal infiltration. Furthermore, with the treatment information, the regions of cornea lesions became more precise and comprehensive. This interpretable feature of our model can further facilitate its application in the real world, as ophthalmologists can understand how the final output of the model is made.

So far, there were insufficient studies on applying deep learning algorithms for infectious keratitis via using slit-lamp images, let alone combined with treatment text. And because of the similarity of BK and FK, no study has had a satisfactory result in this regard. Xu et al. reported an average accuracy of 79% on IK by using a deep sequential-level learning model with slit-lamp images, while their model performed poorly in identifying BK with an accuracy of only 65%^19^. Hung et al. applied segmented images to reach an average diagnostic accuracy of 80% to BK and FK. They used U^2^ Net to crop the image of the cornea because they found inappropriate focusing on the area without clinically relevant features would decrease model performance^20^. Ghosh et al.^22^ applied ensemble learning with three pre-trained CNNs (VGG19, ResNet50 and DenseNet121) that trained on the ImageNet data set and got the best average accuracy of 83% between BK and FK. The above researches were just performed on a single slit-lamp images and their model performed barely satisfactorily in identifying BK and FK. And the performance of all models was closely related to the distribution of the data set. All indicated that there are limitations to using only images as input. And in real world applications, there is more information relevant to diseases, such as medical history, laboratory findings and past history. Hence, we applied image and medication information to improve the model's ability to distinguish BK from FK.

Our model could learn from changes in images between initial and subsequent visits as well as medication intervention. When doctors can’t determine the cause, they would apply to empirical therapy which, if inappropriate, can cause the identifying features to be obscured^11,29^. This in turn would increase patients’ financial burden and may result in a worse prognosis. From Table 1, we concluded that days of the interval were fewer for patients diagnosed with BK than with FK and the difference was meaningful with a *P* value (< 0.001). It is likely caused by the fact that BK progresses more quickly and that doctors tend to monitor the effects of treatment before culture results are known, whereas FK has a longer drug history before culture results are known or symptoms worsen. Thus, reducing the time lag between patient diagnoses not merely lightens the burden on the patient but also decreases the difficulty of microbial keratitis management. In clinical practice, when doctors are unable to diagnose whether it is BK or FK, our model provides a more accurate reference for them to make a more convincing judgement. Moreover, our model has confirmed the potential of multimodality in keratitis.

However, our model has a few limitations. First, we excluded complicated cases, such as patients with mixed infections and other corneal diseases and that would influence the performance of the model. Second, on account of the difficulties of collecting patient records and cleaning images with only a few workers, the size of our dataset was still too small to develop deeper-level experiments. After validating the feasibility of a small data set, it can be extended to a large one according to the user's needs. Third, as we could not match general statistical characteristics of patients (age, gender, etc.) between the training and test groups, changes in these characteristics may have an impact on the model's performance. Finally, the model’s function lies in assisting in the differentiation of FK from BK, and we did not subclassify the dataset to different pathogens, which may have different clinical characteristics. Viral and amoeba keratitis were not included in this study, either. In clinical practice, cultures remain the gold standard for final species identification.

In conclusion, we developed a new deep learning model that combined CNN with BERT to improve the prediction in differentiating between BK and FK. And we are the first study to focus on the impact of image changes and medication interventions on infectious keratitis. Moreover, the method is scalable and can be applied to any clinical problem where the disease is difficult to distinguish based on images but there is other data available in the clinic than images. We believe that the model’s outstanding performance demonstrates the great potential and inspires others of multimodal information for clinical applications.

## Supplementary Information

Supplementary Information.

## Data Availability

The datasets during the current study are not publicly available due to privacy restrictions but are available from the corresponding author upon reasonable request. Interested parties should contact Fei Wu and Yu-Feng Yao. The model source code is available at https://github.com/YuanZhouhang/KTBMC.

## References

[1] Papaioannou L, Miligkos M, Papathanassiou M (2016). Corneal collagen cross-linking for infectious keratitis: A Systematic review and meta-analysis. Cornea.

[2] Ung L, Bispo PJM, Shanbhag SS, Gilmore MS, Chodosh J (2019). The persistent dilemma of microbial keratitis: Global burden, diagnosis, and antimicrobial resistance. Surv. Ophthalmol..

[3] Austin A, Lietman T, Rose-Nussbaumer J (2017). Update on the management of infectious keratitis. Ophthalmology.

[4] Truong DT, Bui M-T, Cavanagh HD (2018). Epidemiology and outcome of microbial keratitis: Private university versus urban public hospital care. Eye Contact Lens.

[5] Khor W-B (2018). The Asia Cornea Society Infectious Keratitis Study: A prospective multicenter study of infectious keratitis in Asia. Am. J. Ophthalmol..

[6] Fleiszig SMJ (2020). Contact lens-related corneal infection: Intrinsic resistance and its compromise. Prog. Retin. Eye Res..

[7] Kuo M-T (2021). Comparisons of deep learning algorithms for diagnosing bacterial keratitis via external eye photographs. Sci. Rep..

[8] Hung N (2020). Filamentous fungal keratitis in Taiwan: Based on molecular diagnosis. Transl. Vis. Sci. Technol..

[9] Dalmon C (2012). The clinical differentiation of bacterial and fungal keratitis: A photographic survey. Invest. Ophthalmol. Vis. Sci..

[10] Ni N (2015). Seasonal, geographic, and antimicrobial resistance patterns in microbial keratitis: 4-Year experience in eastern Pennsylvania. Cornea.

[11] McLeod SD (1996). The role of smears, cultures, and antibiotic sensitivity testing in the management of suspected infectious keratitis. Ophthalmology.

[12] Shah VM (2010). Randomized clinical study for comparative evaluation of fourth-generation fluoroquinolones with the combination of fortified antibiotics in the treatment of bacterial corneal ulcers. Cornea.

[13] Hsu HY (2010). Community opinions in the management of corneal ulcers and ophthalmic antibiotics: A survey of 4 states. Eye Contact Lens.

[14] Qian Y, Meisler DM, Langston RHS, Jeng BH (2010). Clinical experience with Acanthamoeba keratitis at the cole eye institute, 1999–2008. Cornea.

[15] Gulshan V (2016). Development and validation of a deep learning algorithm for detection of diabetic retinopathy in retinal fundus photographs. JAMA.

[16] Ting DSW (2017). Development and validation of a deep learning system for diabetic retinopathy and related eye diseases using retinal images from multiethnic populations with diabetes. JAMA.

[17] Kim SJ, Cho KJ, Oh S (2017). Development of machine learning models for diagnosis of glaucoma. PLoS ONE.

[18] Ting DSW, Lee AY, Wong TY (2019). An ophthalmologist’s guide to deciphering studies in artificial intelligence. Ophthalmology.

[19] Xu Y (2021). Deep sequential feature learning in clinical image classification of infectious keratitis. Engineering.

[20] Hung N (2021). Using slit-lamp images for deep learning-based identification of bacterial and fungal keratitis: Model development and validation with different convolutional neural networks. Diagnostics.

[21] Mayya V (2021). Multi-scale convolutional neural network for accurate corneal segmentation in early detection of fungal keratitis. J. Fungi.

[22] Ghosh AK, Thammasudjarit R, Jongkhajornpong P, Attia J, Thakkinstian A (2022). Deep learning for discrimination between fungal keratitis and bacterial keratitis: DeepKeratitis. Cornea.

[23] Kiela, D., Bhooshan, S., Firooz, H., Perez, E. & Testuggine, D. *Supervised multimodal bitransformers for classifying images and text*. 10.48550/arXiv.1909.02950 (2020).

[24] Devlin, J., Chang, M.-W., Lee, K. & Toutanova, K. *BERT: Pre-training of deep bidirectional transformers for language understanding*. 10.48550/arXiv.1810.04805 (2019).

[25] Deep Residual Learning for Image Recognition | IEEE Conference Publication | IEEE Xplore. https://ieeexplore.ieee.org/document/7780459.

[26] Huang, G., Liu, Z., Van Der Maaten, L. & Weinberger, K. Q. Densely connected convolutional networks. in *2017 IEEE Conference on Computer Vision and Pattern Recognition (CVPR)* 2261–2269. 10.1109/CVPR.2017.243 (2017).

[27] Selvaraju, R. R. et al. *Grad-CAM: Visual explanations from deep networks via gradient-based localization*. 10.1007/s11263-019-01228-7 (2019).

[28] Kuo M-T (2020). A deep learning approach in diagnosing fungal keratitis based on corneal photographs. Sci. Rep..

[29] Gopinathan U (2002). The epidemiological features and laboratory results of fungal keratitis: A 10-year review at a referral eye care center in South India. Cornea.

