# Supplementary material for: A knowledge-enhanced transform-based multimodal classifier for microbial keratitis identification
Source: Sci Rep. 2023 Jun 2;13:9003. doi: 10.1038/s41598-023-36024-4 (PMC10238367; doi:10.1038/s41598-023-36024-4)
Supplement: Supplementary file 1 — Supplementary Information. [file 41598_2023_36024_MOESM1_ESM.pdf]

A Knowledge- enhanced Transform-Based Multimodal Classifier for microbial keratitis identification

Jianfeng Wu[1], Zhouhang Yuan[2], Zhengqing Fang[2], Zhengxing Huang, Yesheng Xu[3,4],Wenjia Xie[3,4], Fei Wu[2,*] and Yu-Feng Yao[3,4,*]

Supplementary Materials

List of Supplementary Materials

Table S1 and Figure S1

Our study has applied the variable learning rate by exponential form of growth ($new\_lr = initial\_lr \times \gamma^{\text{epoch}}$ Equation 1).

$$new\_lr = initial\_lr \times \gamma^{\text{epoch}} \qquad \text{Equation 1}$$

*lr: learning rate

Hyperparameter	Number
Epoch	30
Batch size	16
Initial learning rate	0.001
γ	0.98
Seed	16

[1] School of Public Health, Zhejiang University, Hangzhou 31002, China

[2] College of Computer Science and Technology, Zhejiang University, Hangzhou 31002, China

[3] Department of Ophthalmology, Sir Run Run Shaw Hospital, Zhejiang University School of Medicine, Hangzhou 310016, China

[4] Key Laboratory for Corneal Diseases Research of Zhejiang Province

[*] Corresponding authors.

E-mail addresses: mailto:wufei@zju.edu.cn(F. Wu), mailto:yaoyf@zju.edu.cn(Y.-F. Yao).

Table S 1. Hyperparameter configuration in the experiment

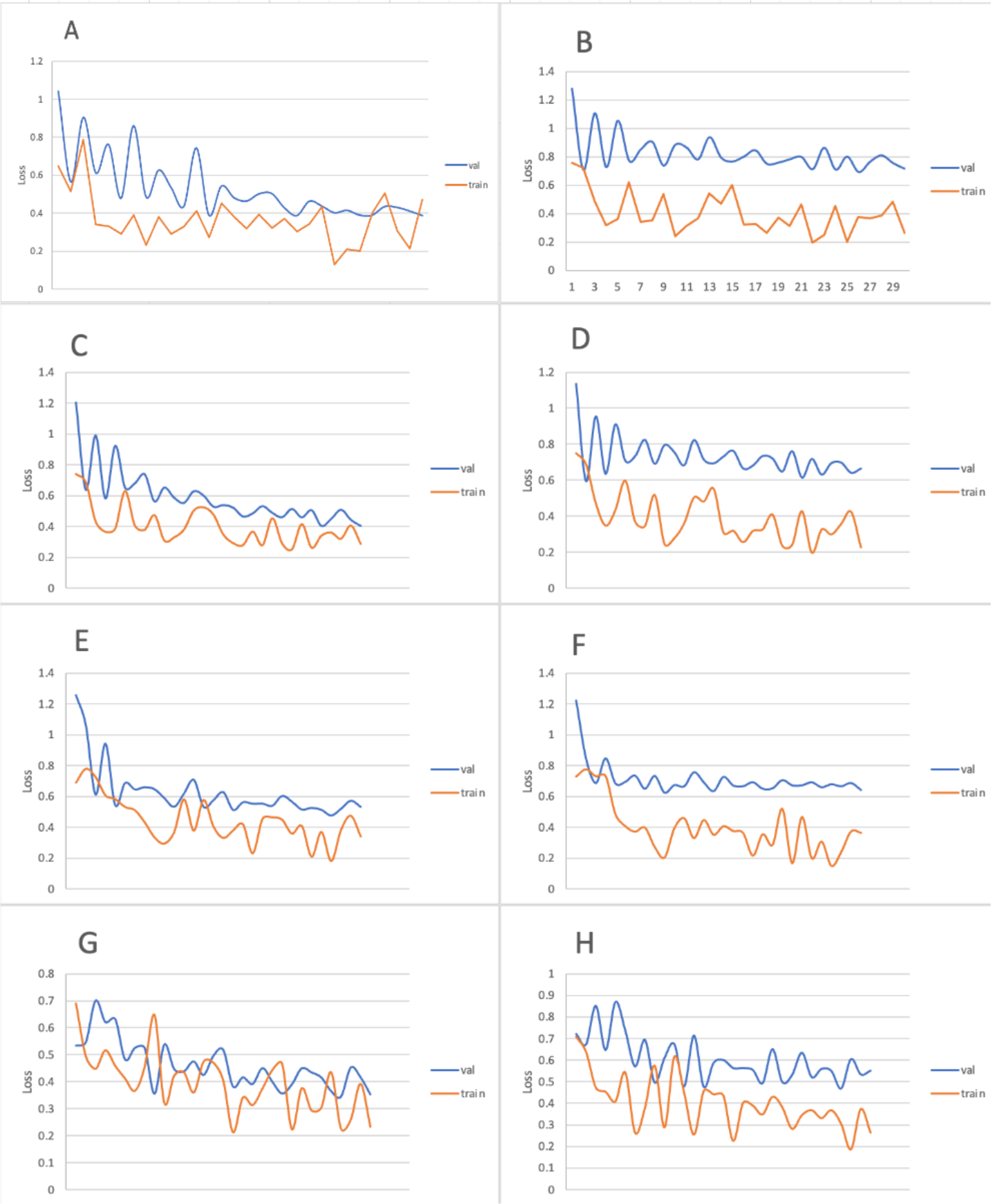

Figure S 1. The loss results of KTBMC with different input and CNN: (A)ResNet152 with text, (B) ResNet152 without text,(C) ResNet50 with text, (D) ResNet50 without text, (E) DenseNet121 with text, (F) DenseNet121 without text, (G) DenseNet169 with text, (H) DenseNet169 without text.
